# Early adolescent perceived friendship quality aids affective and neural responses to social inclusion and exclusion in young adults with and without adverse childhood experiences

**DOI:** 10.1093/scan/nsae044

**Published:** 2024-06-21

**Authors:** Maria R Dauvermann, Laura Moreno-Lopéz, Benedetta Vai, Nadia González-García, Sofia Orellana, Peter B Jones, Ed Bullmore, Ian M Goodyer, Anne-Laura van Harmelen

**Affiliations:** Department of Psychiatry, University of Cambridge, Cambridge, CB2 8AH, United Kingdom; Institute for Mental Health, University of Birmingham, Birmingham, B15 2TT, United Kingdom; Department of Psychiatry, University of Cambridge, Cambridge, CB2 8AH, United Kingdom; Psychiatry & Clinical Psychobiology Unit, Division of Neuroscience, IRCCS San Raffaele Scientific Institute, Milano, 20127, Italy; Department of Psychiatry, University of Cambridge, Cambridge, CB2 8AH, United Kingdom; Laboratory of Neurosciences, Hospital Infantil de México Federico Gómez, Mexico City, 06720, Mexico; Department of Psychiatry, University of Cambridge, Cambridge, CB2 8AH, United Kingdom; Department of Psychiatry, University of Cambridge, Cambridge, CB2 8AH, United Kingdom; Department of Psychiatry, University of Cambridge, Cambridge, CB2 8AH, United Kingdom; Department of Research and Development, Cambridgeshire & Peterborough NHS Foundation Trust, Cambridge, CB21 5EF, United Kingdom; Department of Psychiatry, University of Cambridge, Cambridge, CB2 8AH, United Kingdom; Department of Psychiatry, University of Cambridge, Cambridge, CB2 8AH, United Kingdom; Institute of Education and Child Studies, Leiden University, Leiden, AK 2333, The Netherlands

**Keywords:** friendships, resilient functioning, childhood adversity, social exclusion, functional magnetic resonance imaging

## Abstract

Friendships increase mental wellbeing and resilient functioning in young people with childhood adversity (CA). However, the mechanisms of this relationship are unknown. We examined the relationship between perceived friendship quality at age 14 after the experience of CA and reduced affective and neural responses to social exclusion at age 24. Resilient functioning was quantified as psychosocial functioning relative to the degree of CA severity in 310 participants at age 24. From this cohort, 62 young people with and without CA underwent functional Magnetic Resonance Imaging to assess brain responses to social inclusion and exclusion. We observed that good friendship quality was significantly associated with better resilient functioning. Both friendship quality and resilient functioning were related to increased affective responses to social inclusion. We also found that friendship quality, but not resilient functioning, was associated with increased dorsomedial prefrontal cortex responses to peer exclusion. Our findings suggest that friendship quality in early adolescence may contribute to the evaluation of social inclusion by increasing affective sensitivity to positive social experiences and increased brain activity in regions involved in emotion regulation to negative social experiences. Future research is needed to clarify this relationship with resilient functioning in early adulthood.

## Introduction

Childhood adversity (CA) is the most common cause of mental health problems in children and adolescents growing up worldwide. Such experiences are known to negatively impact on a cascade of functions, including behavioral, cognitive, and interpersonal problems (e.g. poor inhibitory control and emotion regulation, lower self-esteem, higher aggression, and criminality) (Danese et al. 2007, Egeland 2009, Wright et al. 2009, Yates and Wekerle 2009, Majer et al. 2010, Nanni et al. 2012). One of the proposed mechanisms through which CA affects mental health is by increased vulnerability to subsequent psychosocial stress and isolation. Children with a history of CA are more sensitive to further stressful interpersonal interactions (van Harmelen et al. 2014), and are more likely to experience negative peer interactions in adolescence (van Harmelen et al. 2016) than children without such adverse experiences. Therefore, a better understanding of how to enhance resilience to social stress would likely improve mental wellbeing in these vulnerable young people.

Resilience is an umbrella term that has been used to summarize multiple concepts, including the capacity to cope with future stress, an adaptive response to stress or an outcome, which is typically used and refers to the absence of mental health problems in the aftermath of stress (Choi et al. 2019). These three concepts were recently brought together in the “resilience framework” (Kalisch et al. 2017). In this framework, “resilience factors” refer to the stable and dynamic traits that aid ‘capacity’ to respond well to future stress. In contrast, the “resilience mechanism” refers to the ‘process’ of positive adaptation in the face of stress, whereas good mental health and wellbeing in the aftermath of the stressor are captured by “resilient functioning.” For example, a person’s genetic profile (the “resilience factor”) may cause them to show blunted responses during stress (“resilience mechanism”), which result in good mental health and wellbeing after the stressor dissipates (“resilient functioning”).

In the context of CA, where the stressor has previously taken place, resilience can only be examined *ex post facto* in the form of resilient functioning. Such resilient functioning has been quantified as “psychosocial functioning across a range of relevant domains, which is better than in others with similar CA experiences” (van Harmelen et al. 2017, 2021, Ioannidis et al. 2020). Using this approach, individual level of resilient functioning is determined by the degree of better (or worse) psychosocial functioning relative to CA experiences and provides a continuous assessment of individual resilient functioning (see Ioannidis et al. 2020 for benefits and drawbacks of this approach).

Resilient functioning after CA is facilitated by complex interactions of resilience factors that span intrapersonal (e.g. (epi)genetic, biological and cognitive factors) as well as interpersonal levels (cultural resources and social support) (Kalisch et al. 2017, 2019, Ioannidis et al. 2020). Among psychological and social support systems, supportive friendships are especially important since adolescents are highly sensitive to peer influences during early adolescence (approximately 12–14 years of age) (Knoll et al. 2015, 2017, Foulkes et al. 2018, Schreuders et al. 2021). We have recently presented evidence that early adolescent perceived friendships exert a beneficial effect on subsequent resilient functioning (van Harmelen et al. 2017), which was driven by higher quality of friendship support at age 14 (Fritz et al. 2019). Such increased levels of perceived friendship quality are significantly associated with both reduced depressive symptoms (van Harmelen et al. 2016) and better resilient functioning between ages 14 and 17 in adolescents with CA experiences (van Harmelen et al. 2021). However, it is unknown why and how early adolescent friendships aid resilient functioning in adolescents with CA. Here, we used the residual scores for the calculation of the individual degree of resilient functioning to quantify resilient functioning as psychosocial functioning conditional on the degree of CA experiences. This multivariate approach allows us to operationalize “resilient functioning” (Kalisch et al. 2017, 2019, Ioannidis et al. 2020) as the degree to which an individual has better mental health and wellbeing, across mental health domains, and compared to others with similar levels of CA experiences. This method advances the more often used dichotomized quantification of resilience as the absence of mental illness in those with a history of CA in multiple ways. First, it provides a measure of functioning across several measures of mental health and wellbeing. Second, this method considers the subjective interpretation of the severity of early adverse experiences that a person has experienced; such that individuals with similar levels of mental health and wellbeing show different levels of resilient functioning when one has experienced more severe adversity than the other. Finally, this method provides a continuous score for every individual in the sample, allowing for analyses that take a longitudinal approach, leading to more statistical power to detect true effects, which is helpful in smaller samples. This is a well validated method to quantify resilient functioning (e.g. (Bowes et al. 2010, Sapouna and Wolke 2013, Amstadter et al. 2014, van Harmelen et al. 2017, 2021, Veer et al. 2021) and has acceptable psychometric properties (Cahill et al. 2022).

In previous work, we have shown consistently that friendships aid resilient functioning in young people with adverse family experiences during childhood and early adolescence (van Harmelen et al. 2016, 2017, 2021). The “social stress buffering” hypothesis may explain the link between early adolescent perceived friendship support and resilient functioning (Kaplan et al. 1977, Gunnar 2017). This hypothesis states that subjectively available social support ameliorates or “buffers” the potentially negative effects of stressful events (Hennessy et al. 2009, Doom et al. 2017). It has been shown that social support being present during a negative experience, such as a stress task, may act as a “social buffer” by reducing the individual’s stress levels in adolescents (i.e. reducing the cortisol stress response as part of the hypothalamic-pituitary-adrenocortical system) (Gunnar and Hostinar 2015), although it is not well understood what the biological mechanisms of social buffering may be. Indeed, there is preliminary evidence that suggests that friendships reduce stress responses in young people (Masten et al. 2012). Yet, a recent systematic review of the literature showed that only two studies examined social stress buffering in young people with CA and found mixed evidence (Scheuplein and van Harmelen 2022). Therefore, this prospective study in young people with CA experience investigated whether good friendship quality during early adolescence may aid resilient functioning in early adulthood, possibly through social buffering on social exclusion experiences using the Cyberball task.

The Cyberball task is a widely established experimental ostracism task (Williams et al. 2000, Williams and Jarvis 2006) known to induce emotional distress and increased activity in brain regions related to emotion regulation and re-appraisal in healthy young people (Sebastian et al. 2010, Duncan et al. 2019). Using this Cyberball game in healthy young people, studies showed that good quality friendships resulted in lower mood and neural responses to social exclusion in areas of the brain, including the cingulate cortex and insula, that have previously been associated with social cognitive processing (Onoda et al. 2009, Masten et al. 2012). It is possible that these findings may reflect a social buffering effect of adolescent friendships. However, it remains unclear whether perceived friendship quality at age 14 improves resilient functioning experiences in young adults with a history of CA through social stress buffering on a neural level.

The main aim of this study was to investigate whether perceived friendships at age 14 would be related to resilient functioning in individuals reporting CA at age 24, and/or their affective and neural responses to social exclusion at age 24. We tested this aim in 62 young people recruited from a prospective cohort study (ROOTS, *N* = 1238) (Goodyer et al. 2010, Lewis et al. 2016). All participants played the Cyberball game, while undergoing a functional brain scan in the Magnetic Resonance Imaging (MRI) scanner. Based on the available evidence on the role of good quality friendships during the Cyberball game in young people, we hypothesized that in CA vulnerable individuals perceived good friendship quality would be associated with reduced neural responses to social exclusion in the cingulate cortex, prefrontal cortex (PFC), insula, and striatum.

## Material and methods

### Participants and study design

**Figure 1.**
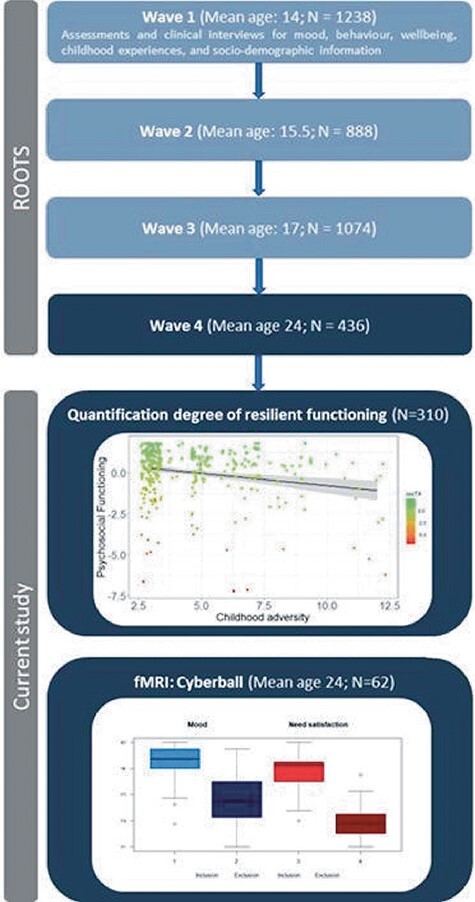
Study overview. Overview of the ROOTS study and additional inclusion criteria for the current study. Resilient functioning at wave 4 was quantified as “resilient functioning relative to the degree of childhood adversity” (van Harmelen et al. 2017). Self-reported mood and need satisfaction after the inclusion and exclusion conditions of the Cyberball task. Abbreviations. fMRI = functional Magnetic Resonance Imaging; resT4 = extracted individual residual scores reflecting the degree of resilient functioning at wave 4.

This study included data from all waves of the longitudinal ROOTS study, a community-based cohort study that followed participants at ages 14, 15.5, 17, and now 24. Full details have been presented previously (e.g. Dunn et al. 2011). Briefly, in this 10-year longitudinal study of youth development, 14 year olds were randomly recruited from 18 schools in the Cambridgeshire and Suffolk counties. Initially, 3762 families received information letters via schools, which introduced the study. ROOTS was approved by the Cambridgeshire and Peterborough Foundation Trust Ethics panel (reference number 03/302) in accordance with the Declaration of Helsinki. All participants and caregivers gave written informed consent and assent at all time points. After providing written informed consent, participants were studied at ages 14 (*N* = 1238), 15.5 (*N* = 888), 17 (*N* = 1074), and 24 (*N* = 436). At these four waves, both self-reports and parent reports on mood, affect, behavior, wellbeing, CA experiences, and sociodemographic information were collected. At age 24, we invited a subsample (*N* = 66, see specifics below) for the current brain imaging study (Fig. 1).

Our main analyses are based on data from the first (age 14) and final (age 24) waves of the ROOTS study. We assessed CA (reported by the caregiver) and quality of perceived friendships (self-reported) in the ROOTS cohort at age 14 and resilient functioning and brain fMRI data in the final wave at age 24.

We employed a double-blind study design with the aim of matching the sample for gender and CA to prevent bias due to CA status at wave 4. To avoid participant and experimenter bias in our study we opted to utilize a double-blind procedure; prior to participation, we did not inform participants that they were invited, in part, because of presence or absence of CA experiences, and the experimenters that interacted with the participant were not aware of the CA status of the participant. Upon completion of the study, however, participants were fully debriefed that CA presence or absence was part of the inclusion criteria and that the experimenter did not know in which group the participant was.

To ensure our sample reflected both low and high levels of resilient functioning at age 24, participants were re-invited to attend the imaging visit at age 24 if they had scored consistently high (>75%) or consistently low (<25%) depressive symptoms [using the Mood and Feelings Questionnaire (MFQ) total score] relative to their gender on two out of the three prior assessment waves (at ages 14, 15.5, and 17). As such, a participant would have been invited if they had >75% highest (or <25% lowest) depressive symptomatology scores, relative to their gender, at ages 14 and 17, or at ages 14 and 15.5. We then subdivided these consistently high or consistently low scoring individuals into those that had experienced any CA in childhood or adolescence or no CA at all (please see Section “Childhood family adversity”). We then ‘invited’ an equal number of high vs low scoring individuals with and without CA to partake in the study. The final sample reflects those that responded to this invitation that did not have the following exclusion criteria (i.e. drug and/or alcohol dependency, learning disability requiring specialist educational support, and the presence of any neurological disorder at age 24 in addition to typical MRI safety exclusion criteria). This led to a final imaging sample of 66 young adults [aged 24, mean = 24.15 years, standard deviation (SD) = 0.71, 38 females]. This sample is described in Table 1 and comparable to the overall ROOTS cohort with no significant differences.

**Table 1.** Demographics and behavioral characteristics of the ROOTS sample at wave 1 (*N* = 1238) and imaging sample at wave 4 (*N* = 62).

	ROOTS sample at wave 1 (*N* = 1238)			Imaging sample at wave 4 (*N* = 62)^g^		
Sex	564 (45.6%) males	674 (54.4%) females		27 (43.5%) males	35 (56.5%) females	
Ethnicity^a^	1087 (93.9%) White	22 (1.9%) Asian	48 (4.2%) Mixed W/B/C/A^b^	57 (96.6%) White	1 (1.7%) Asian	1 (1.7%) Mixed W/B/C/A
	Mean	SD	SE	Mean	SD	SE
Age at interview 1	14.51	0.28	0.01	14.55	0.29	0.04
Friendship quality^c^	23.54	4.21	0.12	22.76	5.18	0.66
CA severity^d^^,^^e^^,^^f^	4.03	1.43	0.04	4.85	2.41	0.31

a Data missing for 81 people for the ROOTS sample at waves 1 and 3 people for the imaging sample at wave 4 (*N* = 62).

b Mixed W/B/C/A = Mixed White, Black, Caribbean, or African.

c Data missing for 105 people for the ROOTS sample at wave 1 (*N* = 1238).

d Classed as 1 = Optimal, 2 = Aberrant, 3 = Discordant, and 4 = Hazardous and added for the periods of 0–6 years, 6–11 years, and 11–14 years. Therefore, the minimum score would be 3 and the maximum score is 12.

e In the imaging sample at wave 4 (*N* = 62), low CA severity levels were observed in 32 young people (51.6%) and high CA severity levels were observed in 30 young people (48.4%).

f Data missing for 98 people for the ROOTS sample at wave 1 (*N* = 1238).

g In the imaging sample at wave 4 (*N* = 62), low depression scores were observed in 33 young people (53.2%) high and depression scores were observed in 29 young people (46.8%). Depression scores were assessed with the MFQ.

Abbreviations. CA, childhood adversity; MFQ, Mood and Feelings Questionnaire; SD, standard deviation; SE, standard error.

In addition, we estimated the individual degree of resilient functioning at age 24 as the individual residual variance of the relationship between psychosocial functioning and CA experiences in the sample; in other words, psychosocial functioning that is better (or worse) than that of others with similar CA experiences in the sample. This residual variance approach to “resilient functioning” (Kalisch et al. 2017, 2019, Ioannidis et al. 2020) was based on individuals with complete data for psychosocial functioning and CA in the final wave (*N* = 310) (please see below for details).

### Assessments

#### Childhood family adversity

Our CA variable is based on an extensive semi-structured, respondent-sensitive interview that collected retrospective accounts of the quality of the family environment from the main caregiver at age 14; the Cambridge Early Experiences Interview (CAMEEI). These recalled experiences were obtained from the main caregiver; biological mothers (96%, *n* = 1143), biological fathers (3%, *n* = 35), with the remaining 1% being divided into: adoptive mothers (*n* = 7), both parents (*n* = 3), and extended family members, step-mothers and step-fathers (*n* = 2) (Dunn et al. 2011). The CAMEEI focuses on three-time domains of childhood: preschool years: early childhood (birth to age 5), late childhood (approximately ages 6–11) and adolescent CA (approximately ages 12–14). Please see supplementary material for details on CAMEEI subtypes.

Because adverse experiences do not occur in isolation; but rather are highly clustered experiences (e.g. see Kessler et al. 2010 for prevalence and overlap between different types of family and extra-family experiences), Dunn et al. (2011) utilized latent class analyses (LCA) to determine whether there were differential dimensions of adverse experiences, or classes, of different early experiences that could be identified in our dataset. This LCA approach of allocating individuals to patterns of family adversities during childhood reflects the exposure to multiple adverse events versus single occurrences that identify exposure of CA over time. Furthermore, for many children, growing up in an adverse family environment spans years during early childhood, later childhood, and early adolescence (∼ 15 years of age). Dunn et al. (2011) found support for four mutually exclusive CA subgroups. The largest class (the “Optimal class”) contained those with a low (<13%) probability of any adversity at any time point (*n* = 784, 69% of the sample). The second class (“Aberrant Parenting”; *n* = 76, 7% of the sample) had a high probability (70–100%) of inconsistent and atypical parenting by both parents (e.g. lax, very strict, cruel to be kind, hitting—all of which showed low prevalence) and a lower probability (8–17%) of any adversity at any time point. The third class (the “Discordant class”; *n* = 213, 19% of the sample) had a high probability (47%) of family discord (e.g. marital disagreements) and 11–39% probability of any adversity at any time points. They also showed elevated rates of family loss, financial difficulties, and maternal psychiatric illness. The fourth class (the “Hazardous class”; *n* = 66, 6% of the sample) had a 50–90% probability of any adversity at any time point, with a high probability (60%) of physical and/or emotional abuse. These classes are ordered according to CA severity with the “Optimal class” reflecting the lowest severity and “Hazardous class” denoting the highest severity level. Each of the four classes was replicated at each time point (birth to age 5; ages 6–11; and ages 12–14) (Dunn et al. 2011).

CA severity was calculated by summing up the class belongingness of the three-time domains of childhood, where a minimum score of 3 denotes belonging to the least severe CA class and a maximum score of 12 represents the most severe CA score across early childhood, late childhood, and adolescence.

#### Perceived friendship quality

Self-reported perceived quality of friendships was assessed with the Cambridge Friendships Questionnaire (CFQ) (Goodyer et al. 1989) at the ages of 14 and 17. The CFQ is an 8-item questionnaire assessing the number, availability, and quality of friendships. Higher scores indicate better perceived overall quality of friendships. The CFQ has good measurement invariance and external validity, and has demonstrated ecological validity across two samples (van Harmelen et al. 2016, 2017). The CFQ has good measurement invariance and external validity, and adequate test–retest reliability across 2 weeks (Kappa = 0.80) (Memarzia et al., unpublished observations). In addition, within the community sample dataset of 2389 healthy adolescents and young adults (aged between 14 and 24 years) as part of the longitudinal Neuroscience in Psychiatry Network (NSPN; http://www.NSPN.org.uk), baseline internal consistency for the CFQ was good (Cronbach’s α = 0.72). Please refer to van Harmelen et al. (2021) for a full version of the CFQ. In a previous study using the entire ROOTS sample, we showed that higher resilient functioning at age 14 in individuals with a history of CA was related to more positive perceived friendship quality increases from age 14 to 17 (van Harmelen et al. 2021).

#### Psychosocial functioning

Degree of psychosocial functioning was based on the MFQ (Angold et al. 1995), Revised Children’s Manifest Anxiety Scale Self-Report questionnaire (RCMAS; Reynolds and Richmond 1997), Short Leyton Obsessional Inventory (LOI; Bamber et al. 2002), and the Child Behaviors Checklist (CBCL) at ages 14, 15.5, 17, and 24.

The MFQ is a 33-item self-report questionnaire that assesses current (past 2 weeks) depressive symptoms. The MFQ has good internal consistency [Cronbach’s α = 0.91; (Thabrew et al. 2018)] and has shown prognostic validity in clinical and non-clinical samples (Wood et al. 1995, Daviss et al. 2006). Higher scores indicate more severe depressive symptoms.

The RCMAS is a 37-item self-report questionnaire that assesses anxiety symptoms. It includes three anxiety factors (physiological anxiety, worry/oversensitivity, and social concerns/concentration), as well as a total anxiety score. Responses range from “always,” “mostly,” “sometimes” to “never.” The internal consistency for the total sum score is excellent [Cronbach’s α = 0.89; (Muris et al. 2002)], and higher sum scores indicate more anxiety symptoms.

The LOI is an 11-item self-report questionnaire that measures obsessional and anxiety symptoms. Responses range from “always,” “mostly,” “sometimes” to “never.” The psychometric properties for the inventory are good [Cronbach’s α = 0.86; (Berg et al. 1988)], and higher scores indicate more obsession and anxiety symptoms.

The original CBCL consists of 113 questions as part of the Achenbach System of Empirically Based Assessment (ASEBA) and measures behavioral and emotional problems in children and adolescents (https://www.apa.org/depression-guideline/child-behavior-checklist.pdf). Here, we used the 11-item self-report questionnaire for symptoms of antisocial behavior based on the Diagnostic and Statistical Manual (DSM-IV) conduct disorder definition. Responses on these items range from “always,” “mostly,” “sometimes” to “never.” These questions to measure conduct disorder have not been previously published. Higher scores indicate more antisocial behavioral symptoms.

### Brain responses to social inclusion and exclusion

Social exclusion in the MRI scanner was assessed with the Cyberball task (Williams et al. 2000, Williams and Jarvis 2006) at age 24. In this task, participants played a game of virtually tossing a ball with two other players (one female and one male) controlled by a computer program. The Cyberball game consists of two parts: the inclusion game and the exclusion game. The game begins with the inclusion game which is characterized by an equal number of ball tossing (i.e. receiving the ball) among players (i.e. one-third of the total ball throws). In contrast, during social exclusion, the participants received the ball only once at the beginning of the game and never again until the end. Both the inclusion and exclusion conditions consisted of a total of 30 ball tosses, and each game was administered in a separate run that lasted approximately 5 minutes. The duration of each ball toss was fixed to 2 seconds. We added a random jitter interval (100–4000 ms) to account for the reaction time of a real player. To further increase credibility of the Cyberball game, both games started with a loading screen that notified that “the computer is trying to connect with the other players.”

#### Affective responses to social inclusion and exclusion

To assess current need satisfaction and mood during the Cyberball game, participants completed a scale developed by Moor et al. (Moor et al. 2012) with eight items from the Need Threat Scale (Williams 2009), which included ratings of self-esteem, belonging, meaningful existence and control (two questions for each need), and eight items from a mood questionnaire (feeling good/bad, happy/sad, relaxed/tense, and friendly/unfriendly) (Sebastian et al. 2010). Items on these questionnaires were rated from 1 (“not at all”) to 5 (“very much”), with high scores indicating good mood or greater need threat (i.e. low self-esteem, low sense of belonging, low sense of meaningful existence, and low sense of control). Importantly, to enhance the readability of this paper, we inverted the need threat scores such that high scores indicated higher self-esteem, greater sense of belonging, higher meaningful existence, and better control. To examine the affective responses of social exclusion (vs inclusion), we calculated change scores by subtracting mood and need satisfaction scores reported after the inclusion condition from the exclusion condition. Thus, the obtained values indicated the change in mood and/or need satisfaction in response to social exclusion (relative to social inclusion). Please see the supplementary material for details.

### Image acquisition and pre-processing

In the imaging cohort, *N* = 66, most of the participants (*N* = 51; 26 females) were scanned at the Cognition and Brain Sciences Unit (CBU) in Cambridge, using a 3T Siemens Prisma MR system with a 32-parallel transmit head coil. The remainder of the participants (*N *= 15, 12 females) were scanned at a Siemens Trio 3-Tesla MR system at the Wolfson Brain Imaging Centre of Addenbrooke’s Hospital (Cambridge, UK) with a standard 32-channel radiofrequency (RF) head coil. At both scanners, the imaging session started with a localizer followed by a structural scan using a multi-parametric mapping (MPM) protocol. Finally, an echo-planar imaging (EPI) sequence with the following parameters was acquired: TR = 2000 2000ly, an echo-planar gle = 78°, FOV = 220 mm, voxel size = 2.8 × 2.8 × 2.8 mm, slices per volume = 36. As a proportion of subjects was imaged at a different scanner with different sequences, we analyzed if the different scanning sites influenced the EPI scans. Therefore, the mean and SD of the time series across the 308 ROI were determined. We calculated the coefficient of variation (mean/SD) for each participant. Mean and coefficient of variation maps were generated for visual assessment. Finally, we conducted a t-test to compare both sites, which was not significant (t_(22.54)_ = –1.39, *P* = .179).

Data were pre-processed and analyzed using Statistical Parametric Mapping (SPM12; Wellcome Department of Cognitive Neurology, London), running in MATLAB R2016a. The setup, acquisition, and post-processing of the structural images have been described elsewhere (Weiskopf et al. 2013). EPI scans were first reoriented to the anterior and posterior commissures (ac/PC). Outlier images with excessive motion (>1 mm, >0.02 rad) or spikes in global signal intensity (>3 SD from mean) were identified using Artifact Detection Tools (ART; http://www.nitrc.org/projects/artifact_detect). From our original sample of 66 participants, we excluded four subjects due to excessive head movement. The remaining 62 MRIs were then corrected for differences in timing of slice acquisition, rigid body motion correction, normalized to the International Consortium of Brain Mapping (ICBM) brain template (ICBM’152), a standard EPI template volume based on the Montreal Neurological Institute and smoothed using an 8-mm full-width at half-maximum isotropic Gaussian kernel.

### Behavioral analyses

Analyses were conducted in R (version 4.0.2) (Team 2020).

#### Calculation of resilient functioning

Resilient functioning was calculated for all participants of the final wave (wave 4; age 24) of the ROOTS study (*N* = 436). The imaging cohort filled in the questionnaire at home and during the assessment just before the scanning. For this reason, we utilized the scores from the day of scanning for the imaging participants.

We calculated resilient functioning in line with our previous work (van Harmelen et al. 2017, 2021). Specifically, we quantified individual resilient functioning as the individual residual variance of the relationship between psychosocial functioning and CA experiences; the extent to which a person is functioning better or worse than expected to others with similar CA levels in the sample. This is a well validated method to quantify individual degree of risk to resilient functioning (e.g. (Bowes et al. 2010, Sapouna and Wolke 2013, Amstadter et al. 2014, van Harmelen et al. 2017, 2021, Veer et al. 2021) and has good psychometric properties (Cahill et al. 2022). This conceptualization better separates psychosocial functioning especially towards the extremes of CA severity than more conventional resilience examinations in cross-sectional neuroimaging research, such as the most often used group comparison approach that examines traumatized individuals with vs without psychopathology (Moreno-López et al. 2020). The residual approach further considers individual degree of psychosocial functioning and CA severity and provides each individual in the sample with a single continuous score that represents the extent to which an individual’s psychosocial functioning is better or worse than expected given their CA experiences (for more information about this approach please see Kalisch et al. 2017, 2019, Ioannidis et al. 2020). However, the residual method also entails a *a priori* strong association between resilient functioning and the measures of psychosocial functioning; the residuals will be highly correlated with the psychosocial functioning outcomes on which they are based, especially in very resilient samples, or samples reporting less severe CA. Therefore, the residuals will depend on, and only relate to, the sample used to construct them, and as such should ‘only’ be interpreted as current resilient (psychosocial) functioning.

Resilient functioning was calculated for the sample of individuals that had full data on the below questionnaires (*N* = 310). In this sample, we used a principal component analysis to calculate individual level of psychosocial functioning as the first step by using scaled sum scores for the MFQ, RCMAS, LOI, and CBCL. This principal component analysis revealed that a first factor explained 65% variance across these measures and had positive factor loadings (MFQ = 0.57, RCMAS = 0.59, LOI = 0.49, CBCL = 0.29), suggesting that a higher factor score was indicative of worse psychosocial functioning. For the below analyses, we extracted these individual factor scores for the entire sample (*N* = 310) and inverted them such that positive scores indicate better psychosocial functioning. Second, to calculate individual resilient functioning scores, individual accumulated CA scores were regressed on the individual factor score for psychosocial functioning. This analysis revealed a significant negative relationship between CA and psychosocial functioning [F_(1304)_ = 14.47, R^2^ adj = 0.042, *P* < .001; coefficient: Estimate (Est) = –0.151, SE = 0.04, t = –3.804, *P* < .001; Fig. 1]. We note that within the Cyberball imaging sample (*N* = 62) a *post-hoc* linear regression also revealed a significant negative relationship between CA and psychosocial functioning [F_(1,60)_ = 5.505, R^2^ adj = 0.068, *P* = .022; coefficient: Estimate (Est) = –0.3722, SE = 0.159, t = –2.346, *P* = .022; Supplementary Fig. S2a]. Therefore, in the next step, and using the model based on the entire dataset (*N* = 310), we extracted residual scores from the relation between CA and psychosocial functioning for the 62 participants in the Cyberball imaging study. These residual scores reflect how much better or worse an individual is functioning compared to the mean average of psychosocial functioning of those in the larger sample with similar CA experiences. In other words, the extracted individual residual scores provide an indication of the individual degree of resilient functioning at age 24 relative to the severity of CA experienced in our sample.

#### Imaging analyses

Blood oxygen level-dependent (BOLD) responses were distinguished for the two conditions of the Cyberball game: inclusion (i.e. participants received the ball throughout the complete game) or exclusion (i.e. participant received the ball only once at the beginning of the game). For the first-level analysis, the evoked responses were modeled as events in a design matrix comprising the onset of the stimuli. The regressors modeling events were then convolved with a canonical hemodynamic response function (HRF), spatial and temporal derivates, the six motion parameters, and parameter estimated for all regressors. The time series across each voxel were high-pass filtered to 1/128 Hz, and serial autocorrelations were modeled as an AR(1) process. We defined three conditions of interest: “receiving the ball,” “playing the ball,” and “not receiving the ball.” To examine the effects of social exclusion, we compared brain responses using the t contrast: “Not receiving the ball during the exclusion condition vs Receiving the ball during the inclusion condition.” Our imaging analyses were performed at the pFWE < 0.05 whole-brain level, both looking at the main effect of the task and the correlations between the activation found in the task and our variables of interest (i.e. perceived friendship quality at age 14 and age 17, resilient functioning at 24 and CA). Significant findings were followed up with a robust regression using Huber weights and a sensitivity analysis by excluding outliers based on large residuals and/or high leverage for perceived friendship quality and resilient functioning scores as identified by Ordinary Least Squares (OLS) to ensure the significance of the observed associations. In addition, in *post-hoc* analyses, we explored whether any cluster activation findings for the dorsomedial prefrontal cortex (DMPFC) related to perceived friendship quality at age 14 were confounded by perceived friendship quality at age 17 or CA experiences.

#### Statistical analyses

To investigate whether perceived friendship quality at age 14 would be related to resilient functioning in individuals reporting CA at age 24, we ran a linear regression with perceived friendship quality at age 14 as predictor and resilient functioning scores at age 24 as outcome. Finally, in *post-hoc* analyses, we investigated whether this relationship was distinct to perceived friendship quality in ‘early’ adolescence, or whether it was also found for perceived friendship quality at age 17. Therefore, we re-ran the model with (i) perceived friendship quality at age 17 as a covariate, and (ii) perceived friendship quality at ‘age 17’ as predictor of resilient functioning at age 24.

Next, we investigated associations between perceived friendship quality at age 14, resilient functioning at age 24, and change in mood and need satisfaction from inclusion to exclusion using linear regressions. In all analyses, we added age and gender as covariates. Findings were considered significant at *P *< .05 two-sided.

## Results

### Further study characteristics on mood and need satisfaction during the Cyberball task, psychosocial functioning, and resilient functioning

Demographic details, study characteristics, and CA of the imaging sample are reported in Table 1. Data on mood and need satisfaction during the Cyberball task, psychosocial functioning, and resilient functioning are provided in Table 2.

**Table 2.** Affective and behavioral characteristics of the imaging sample at wave 4 (*N* = 62).

	Mean	SD	SE
Age at scanning	24.13	0.71	0.09
Psychosocial functioning	−0.04	1.88	0.24
Resilient functioning	−0.02	1.81	0.23
Mood at inclusion	4.27	0.64	0.08
Mood at exclusion	2.83	0.90	0.11
Need satisfaction at inclusion	3.89	0.63	0.08
Need satisfaction at exclusion	1.90	0.59	0.08
Mood change	1.43	0.94	0.12
Need satisfaction change	2.0	0.67	0.09

Notes.

Resilient functioning was quantified as psychosocial functioning relative to the degree of CA severity at age 24, whereas psychosocial functioning refers to measures of depressive, anxiety, obsessional, and antisocial behavior symptoms assessed at ages 14, 15.5, 17, and 24.

Mood change and need satisfaction change refer to the response to social exclusion relative to social inclusion during the Cyberball task.

Abbreviations. SD, standard deviation; SE, standard error.

### Association between adolescent perceived friendship quality at ages 14 or 17 and resilient functioning at age 24

We found that perceived good friendship quality at age 14 was significantly related to better resilient functioning at age 24 (F_(1,60)_ = 5.256, R^2^ adj = 0.065, *P* < .0254; coefficient: Est = 0.099, SE = 0.04, t = 2.293, *P* = .025). *Post-hoc* analyses revealed that friendship quality at age 14 and friendship quality at age 17 were significantly related (F_(1,58)_ = 32.58, R^2^ adj = 0.3487, *P* < .001; coefficient: Estimate (Est) = 0.770, SE = 0.1349, t = 5.708, *P* < .001). In addition, perceived good friendship quality at ‘age 17’ was also found to be significantly related to better resilient functioning at age 24 (F_(1,50)_ = 17.48, R^2^ adj = 0.2443, *P* < .001; coefficient: Est = 0.1790, SE = 0.0428, t = 4.181, *P* < .001). As such, both friendship qualities at 14 and at 17 were predictive of resilient functioning at age 24. Therefore, in the below analyses, we explored whether perceived friendship quality at 17 confounded any relations between perceived friendship quality at 14 and neural responses to social exclusion.

### Affective responses (mood and social needs satisfaction) to social inclusion and exclusion

As expected, mood and social needs satisfaction were significantly reduced after the exclusion condition (mood; Mean = 4.27, SD = 0.64; need satisfaction, Mean = 3.90, SD = 0.63) when compared to the inclusion condition (mood; Mean = 2.83, SD = 0.90; need satisfaction, Mean = 1.90, SD = 0.60) in all participants (mood; t_(61)_ = 11.953, *P* *< *.001; need satisfaction, t_(61)_ = 23.341, *P* *< *.001) (Fig. 1; fMRI Cyberball). This means that participants showed lower mood and lower social needs satisfaction (i.e. lower self-esteem) during the exclusion condition in contrast to the inclusion condition.

#### Association between resilient functioning and affective responses to social inclusion and exclusion

**Figure 2.**
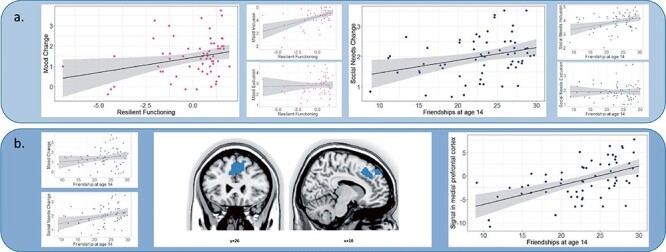
Significant results found in the analyses of the correlations between friendship at 14, resilient functioning at age 24, brain responses to social exclusion, and mood and need satisfaction scores. (a) Association between resilient functioning at age 24 and mood change scores (left panels) and between friendships at 14 and need satisfaction change scores (right panels) across both inclusion and exclusion conditions of the Cyberball task. (b) Association between friendship at 14 and need satisfaction scores across both inclusion and exclusion (left panel), brain responses to social exclusion (middle panel) and plot of the correlation (left panel). Peak coordinates were located at the right dorsomedial prefrontal cortex.

Higher resilient functioning scores at age 24 were associated with more positive mood change from inclusion to exclusion (F_(3,58)_ = 3.078, R^2^ adj = 0.093, *P* = .034; coefficient: Est = 0.1147, SE = 0.07, t = 1.684, *P* = .10). Exploratory *post-hoc* analyses showed that this finding was driven by a significant association between higher resilient functioning and more positive mood scores at inclusion (F_(3,58)_ = 6.212, R^2^ adj = 0.204, *P* < .001; coefficient: Est = 0.147, SE = 0.04, t = 3.377, *P* = .001), but not at exclusion (F_(3,58)_ = 1.085, R^2^ adj = 0.004, *P* = .363) (Fig. 2a).

We similarly observed that higher resilient functioning scores at age 24 and higher change scores for social needs satisfaction were significantly related (F_(3,58)_ = 5.025, R^2^ adj = 0.165, *P* = .004; coefficient: Est = 0.138, SE = 0.05, t = 2.958, *P* = .004) (Fig. 2b). Exploratory analyses revealed that this relationship was driven by a significant association of more positive social needs satisfaction at inclusion (F_(3,58)_ = 4.229, R^2^ adj = 0.137, *P* = .009; coefficient: Est = 0.127, SE = 0.04, t = 2.877, *P* = .006), but not at exclusion (F_(3,58)_ = 0.2481, R^2^ adj = –0.038, *P* = .862).

#### Association between adolescent perceived friendship quality and affective responses to social inclusion and exclusion

More positive friendship quality at age 14 was significantly related to more positive social needs satisfaction change scores at age 24 (F_(4,57)_ = 2.92, R^2^ adj = 0.112, *P* = .029; coefficient: Est = 0.04, SE = 0.02, t = 2.363, *P* = .02), and exploratory analyses revealed that this effect was driven by more positive social needs satisfaction at inclusion (F_(4,57)_ = 3.00, R^2^ adj = 0.116, *P* = .026; coefficient: Est = 0.04, SE = 0.01, t = 2.556, *P* = .013), but not at exclusion (F_(4,57)_ = 0.726, R^2^ adj = –0.018, *P* = .578) (Fig. 2a). When we added perceived friendship quality at age 17 to the regression model, we observed that the positive relationship between better friendship quality at age 14 and social needs satisfaction change scores was no longer significant (F_(2,49)_ = 2.87, R^2^ adj = 0.068, *P* = .066; coefficient: Est = 0.03, SE = 0.02, t = 1.185, *P* = .242). We did not find a significant relationship between perceived friendships at age 14 and mood change in response to social exclusion (Supplementary Fig. S3; please see supporting information for details).

### Brain responses to social inclusion and exclusion

Brain activation for the contrast “Not receiving the ball during the exclusion condition vs. Receiving the ball during the inclusion condition” at pFWE <0.05 comprised a cluster including the right insula and rolandic operculum (peak at x, y, z = 40, −14, 24, t = 6.29, pFWE < 0.05) (Supplementary Fig. S4). No significant activations were found in the contrast “Receiving the ball during the inclusion condition vs Not receiving the ball during the exclusion condition” at pFWE < 0.05.

#### Association between brain responses to social exclusion and perceived friendship quality

Perceived friendship quality at age 14 was significantly associated with DMPFC responses to social exclusion (DMPFC peak at x, y, z = 10, 26, 40; t = 5.70; pFWE < 0.05 at the whole brain level; Fig. 2b). This correlation was followed up with both a robust regression using Huber weights (*N* = 62) and a sensitivity analysis by excluding three outliers based on large residuals of perceived friendship quality as identified by OLS (*n* = 59), where residuals refer to the difference between the observed and predicted values. We observed that the relationship between the DMPFC cluster activation to social exclusion and perceived friendship quality assessed at age 14 (F_(1,60)_ = 12.67, R^2^ adj = 0.1606, *P* < .001; coefficient: Est = 0.4524, SE = 0.1271, t = 3.56, *P* < .001) remained significant following a robust regression analysis (coefficient: value = 0.4436, SE = 0.1285, t = 3.4524) and a sensitivity analysis (F_(1,57)_ = 18.29, R^2^ adj = 0.2297, *P* < .001; coefficient: Est = 0.5715, SE = 0.1336, t = 4.277, *P* < .001).

Perceived friendship quality at age 17 was not significantly associated with any activations at pFWE < 0.05 at the whole brain level. Furthermore, all above results for perceived friendship quality at age 14 remained when friendship quality at 17 and CA were added as covariates to the regression model. Furthermore, *post-hoc* linear regressions revealed that DMPFC activity was not related to perceived friendship quality at age 17 [F_(1,58)_ = 0.063, R^2^ adj = –0.0161, *P* = .8032; coefficient: Estimate (Est) = 0.02882, SE = 0.25, t = 0.803, *P* = .8032], or CA experiences [F_(1,60)_ = 0.5904, R^2^ adj = –0.007, *P* = .4453; coefficient: Estimate (Est) = 0.049, SE = 0.065, t = 0.768, *P* = .445].

#### Association between brain responses to social exclusion and resilient functioning

Resilient functioning at age 24 was not related to brain responses to social exclusion, at the whole brain level pFWE < 0.05. Of note is that, at a lower significance threshold (*P* < .001, k = 10), resilient functioning at age 24 showed an association with DMPFC activation to social exclusion (peak at x, y, z = –14, 26, 30; t = 3.78; *P* < .001, uncorrected).

## Discussion

The aim of this prospectively driven study was to examine whether social stress buffering is a potential explanation for the link between perceived good quality early adolescent friendship support and resilient functioning in early adulthood in individuals with CA experiences. In keeping with previous studies, we hypothesized that perceived friendship support would be related to later resilient functioning. We further assumed that social exclusion (vs inclusion) would lead to lower affective responses (i.e. lower mood and lower need satisfaction) (Sebastian et al. 2010) and reduced neural responses to social exclusion exerting a blunted stress response (Alvares et al. 2010). Furthermore, we hypothesized that perceived friendship quality at age 14, and resilient functioning at age 24, would be associated with ‘reduced’ affective and neural responses to social exclusion at age 24.

In line with these assumptions, we found that perceived friendship quality at age 14 predicted more resilient functioning at age 24. We also found that exclusion (vs inclusion) in the Cyberball task significantly lowered mood and need satisfaction. However, in contrast to our assumptions, we found that perceived friendship quality at age 14 and resilient functioning at age 24 were both related to more ‘positive’ affective responses (i.e. better mood) to social ‘inclusion’. When examining neural responses to social exclusion (vs inclusion), we found that perceived friendship quality at age 14 (but not age 17) was associated with ‘increased’ neural responses in the DMPFC to social ‘exclusion’ (vs inclusion) at age 24. This finding was not in keeping with our hypothesis. Resilient functioning at age 24 was not associated with altered neural responses to social exclusion (vs inclusion). Thus, although perceived friendship support at age 14 was related to affective and neural responses to social exclusion at age 24, we did not find support that this mechanism was directly related to individual degree of resilient functioning at age 24.

### Relationship between perceived good quality friendships and resilient functioning

The role of peer friendship support on young people’s mental wellbeing has been consistently demonstrated (Coverdale and Long 2015, Gunnar and Hostinar 2015, Harding et al. 2015), and our finding of an association between perceived good quality friendship support at age 14 and resilient functioning at age 24 is in keeping with these findings. We have shown that perceived quality friendship support in adolescents reduces depressive symptoms in young people who have been exposed to early life stress (childhood family adversity and/or relational bullying before the age of eleven) (van Harmelen et al. 2016). This suggests that perceived good quality friendship support at age 14 in this study may reflect a mitigating effect on CA experiences expressed in resilient functioning at age 24.

However, despite this link, resilient functioning at age 24 was not associated with DMPFC activation to social exclusion. As such, we found no support for a specific neural mechanism that could explain this relationship in our sample. Future studies may reveal further mechanisms that may underlie the complex relationship between early adolescent friendships and resilient functioning as well as better mental health during early adulthood. More recently, we found preliminary support that perceived young adult friendship quality was associated with lower hippocampal responsivity to acute stress (using the Montreal Imaging Stress Task) in a comparably small sample of young adults (König et al. 2023). These effects were found only in young adults who self-reported early adverse experiences. As such, it should be examined whether friendship buffering effects generalize or extend to other neural stress mechanisms or dimensions of adverse experiences.

### Brain responses to social exclusion

Our findings of neural responses to social exclusion in the insula and the relationship between DMPFC activation to social exclusion and friendship quality in our sample overlap with findings from studies showing a positive relationship between self-reported distress during social exclusion and activity in brain regions associated with affective and pain processing, including the dorsal anterior cingulate cortex, amygdala, periaqueductal grey, and anterior insula (Eisenberger et al. 2003, 2007, Eisenberger 2012, 2013, 2015). We note, however, that we did not find significant correlations between neural responses and resilient functioning in our sample. It remains to be investigated to what extent physical and social pain responses share similar or overlapping neurobiological systems in individuals who have experienced low to severe CA.

When examining the effects of early friendship quality at age 14, we found ‘increased’ DMPFC activation to social exclusion (vs inclusion), which was against our hypothesis of ‘reduced’ activation to social exclusion. This finding is of interest given previous findings showed that increases in gray matter volume of the medial PFC and other metrics predicted friendship quality change over time in adolescents and young adults (Becht et al. 2020). It is widely accepted that the DMPFC is involved in evaluating others’ mental states in relation to outcomes that affect one’s wellbeing, and DMPFC activity therefore may reflect appraisal function (Dixon et al. 2017). Moreover, the DMPFC is highly connected with the temporoparietal junction and temporopolar cortex, which are regions often referred to as the “mentalizing network” (Sebastian et al. 2010). Brain network interactions between these regions may allow individuals to focus on the perspective of others and use social knowledge to infer their mental states (Petrides and Pandya 2007, Zahn et al. 2007, Van Overwalle 2009, Van Overwalle and Baetens 2009, Santiesteban et al. 2012, Andrews-Hanna et al. 2014, Kestemont et al. 2015, Crone and Fuligni 2020). Here, we suggest that DMPFC activity in young people with low to severe CA experiences may reflect emotion regulation or re-appraisal processes, which are based on the judgment of other people’s intentions (i.e. why the other player may or may not toss the ball) (Dixon et al. 2017). In summary, the relationship between DMPFC activity and perceived friendship quality at age 14 may underlie enhanced emotion regulation or (re-appraisal of others’) following social exclusion in young people who have experienced low to severe CA. Perhaps, perceived good quality friendships improve adaptive emotion regulation in response to social exclusion through increased DMPFC activity. However, DMPFC peak activity in our sample was not related to resilient functioning, as such we did not find evidence that increased DMPFC activity acts as a mechanism that directly aids resilient functioning in early adulthood. Future studies are needed to shed light on whether and how perceived friendship quality increases emotion regulation capacity, and what the (in)direct relationship is with resilient functioning in order to optimize ongoing psychotherapeutic and cognitive behavioral interventions.

### Methodological considerations

The residual approach provides estimates of variance from a population specific relationship between CA and mental health and wellbeing. This estimation is limited to the sample in which the analyses are conducted. Therefore, we estimated the resilience scores at age 24 using the largest available sample (*N* = 310) and extract these scores for our imaging sample (*N* = 62), and continuously refer to them as “resilient functioning.” By design, the residual approach assumes a strong relationship between mental health and wellbeing (psychosocial functioning across different measures of depressive, anxiety, obsessional, and antisocial behavior symptoms) and resilient functioning (i.e. the residuals of the relationship between psychosocial functioning and childhood adverse experiences). As such, in a general population sample, such as ROOTS, with low levels of CA experiences, one would expect there to be a small to moderate relationship between CA and mental health and wellbeing. Indeed, in our imaging sample, there was only a moderate relation between CA and psychosocial functioning (r =–0.290, *P* < .022, *n* = 62), as such our measures of psychosocial and resilient functioning are indeed highly correlated (r = 0.981, *P* < .001, *n* = 62). Therefore, our findings related to resilient functioning will be similar to any analysis focusing on psychosocial functioning in our sample that reported low to moderate CA and to those that only examine mental health and wellbeing across domains without taking CA levels into account as previously reviewed (Teicher et al. 2022).

### Limitations

The findings of this study should be considered in light of some limitations. Firstly, not all variables were measured at the same time point, e.g. mood and social needs satisfaction scores, neural activation, and resilient functioning were assessed at age 24. Secondly, perceived friendship quality at age 17 also adjusted the relationship between friendship quality at age 14 and DMPFC activation to social exclusion. However, we acknowledge that this interpretation is preliminary and prone to type 1 errors given the nature of the *post-hoc* regression and correlation analyses, the small sample size, and the lack of a perceived friendship quality measure at age 24. Further research is needed to examine the stability of good quality friendships throughout adolescence and whether the specific phase of adolescence (i.e. early, middle or late adolescence) may act as a social stress buffer with potential long-term consequences for resilient functioning and well-being. Thirdly, a longer follow-up period beyond the 10 years of the presented findings will be useful given the known risk period for the development of mental health conditions until the age of approximately 30 years (Lee et al. 2014). Further studies should examine the differential effects of type and timing of CA (i.e. during early childhood versus adolescence) which may lead to greater insights of differential developmental processes. Finally, these findings need to be replicated in larger samples.

## Conclusion

In conclusion, we provided novel evidence of the beneficial effect of early adolescent perceived friendships on brain responses to social exclusion in early adulthood in young adults with experiences of low to severe CA during childhood or adolescence. These findings offer a preliminary understanding into the potential affective and neurocognitive developmental processes of perceived friendship support in early adolescence. However, future studies are needed to examine the pathways through which friendships during this sensitive developmental period aid later resilient functioning in early adulthood. The potential benefit of peer friendships on mental wellbeing is particularly remarkable given the plethora of evidence suggesting the detrimental role of CA experiences for the development of mental health conditions, such as major depressive disorder, psychosis, suicidal thoughts, and behaviors (Scott et al. 2012, Bonoldi et al. 2013, Hughes et al. 2017, Copeland et al. 2018) as well as greater clinical expression and prevalence of co-existing physical and mental health conditions (Norman et al. 2012, Kalmakis and Chandler 2015). Future studies should investigate whether enhancing peer support is an effective intervention strategy for and with young people with mental health difficulties. Future research is needed to identify whether quality friendship support benefits all young people who have experienced adverse events in their childhood or a specific subgroup with potentially additional vulnerabilities. In particular, future efforts should focus on young individuals at high risk of developing a mental health condition (i.e. subclinical or prodromal symptoms of depression and anxiety) (Heim and Nemeroff 1999, Heim and Binder 2012, Bonoldi et al. 2013, Duffy et al. 2020).

## Supplementary Material

nsae044_Supp

## Data Availability

The data and code will be available upon request from the Cambridge University data repository (https://www.repository.cam.ac.uk/).
