# Supplementary material for: Early adolescent perceived friendship quality aids affective and neural responses to social inclusion and exclusion in young adults with and without adverse childhood experiences
Source: Soc Cogn Affect Neurosci. 2024 Jun 21;19(1):nsae044. doi: 10.1093/scan/nsae044 (PMC11219303; doi:10.1093/scan/nsae044)
Supplement: nsae044_Supp [file nsae044_supp.zip › scan-22-234-File006.docx]

Supplementary Material

# Methods

## Childhood family adversity

Adversities reported in the CAMEEI were as follows:

1. Negative family relationships: (i) family loss and separations (includes step-parents and siblings and partners resident for more than 6 months) through divorce, death or adoption; (ii) family discord; (iii) lack of maternal affection/engagement with the proband; (iv) maternal parenting style; (v) paternal parenting style.
2. Family illness: (i) lifetime family medical illnesses sufficiently severe to impact on family life (moderate, chronic and life-threatening); (ii) lifetime psychopathology in family members.
3. Family Economics: (i) periods of unemployment; (ii) financial difficulties.
4. Childhood Maltreatment: (i) physical abuse; (ii) sexual abuse; (iii) emotional abuse. Including ‘at risk’ children defined as those ever having been on the Child Protection Register or for whom there was strong, but inconclusive, evidence of abuse.
5. Other Events: (i) criminality among family members; (ii) acute life events; (iii) chronic social difficulties (e.g., ongoing litigation or the demands of caring for extended family).

## Need-Threat-Scale and mood questionnaire

Participants completed the questions immediately after each block (whilst lying in the scanner). The Need-Threat-Scale consists of eight items that measure self-esteem, belonging, meaningful existence, and control (each was measured with two questions) (items 1-8). A high score on this scale indicates that the basic needs are threatened (i.e., low self-esteem, low sense of belonging to others, low sense of meaningful existence, and low sense of control). The mood scale consisted of eight items that (two of each) measured feeling good/bad, relaxed/tense, happy/sad, and friendly/unfriendly (items 9-16). All items on the questionnaires were rated from 1 (‘not at all’) to 5 (‘very much’), and a high score on this questionnaire indicates good mood). To enhance the readability of this paper, we inverted the need threat scores (in the original scale, a high need threat score indicated low need threat). We have provided a screenshot of the items, where ‘R’ refers to reverse coded items (Figure S1).

# Results

## Relationship between perceived friendship quality and mood scores

We did not find a significant relationship between perceived friendship quality and mood scores. We observed a nonsignificant association between perceived friendship quality and mood change scores (F_(4,57)_ = 1.75, R^2^ adj = 0.047, p = 0.152; Figure S3A). However, we found a significant relationship between perceived friendship quality and mood scores at inclusion (F_(4,57)_ = 2.678, R^2^ adj = 0.099, p < 0.05; coefficient: Estimate (Est) = 0.03, SE = 0.02, t = 1.774, p = 0.08; Figure S3B). Furthermore, there was no significant relationship between perceived friendship quality and mood scores during exclusion (F_(4,57)_ = 0.800, R^2^ adj = -0.013, p = 0.530; Figure S3C).

**2.2 Brain responses to social exclusion**

Brain activation for the contrast ‘*Not receiving the ball during the exclusion condition vs. Receiving the ball during the inclusion condition*’ comprised a cluster including the right insula and rolandic operculum (Figure S4). No significant activations were found in the contrast *‘Receiving the ball during the inclusion condition vs. Not receiving the ball during the exclusion condition’* at pFWE < 0.05.

**Figures**

**Figure S1. Need Threat Scale and mood questionnaire**

| **1. During the game, I felt like I belonged.** | **1 2 3 4 5** |
| --- | --- |
| **2. I felt accepted by the other players.** | **1 2 3 4 5** |
| **3. I felt that the other players were in control during the game. (R)** | **1 2 3 4 5** |
| **4. I felt that I was in control during the game.** | **1 2 3 4 5** |
| **5. I felt good about myself during the game** | **1 2 3 4 5** |
| **6. My self-esteem was high during the game.** | **1 2 3 4 5** |
| **7. I felt invisible during the game. (R)** | **1 2 3 4 5** |
| **8. I felt that my existence was meaningful during the game.** | **1 2 3 4 5** |
| **9. I felt good during the game.** | **1 2 3 4 5** |
| **10. I felt bad during the game. (R)** | **1 2 3 4 5** |
| **11. I felt happy during the game.** | **1 2 3 4 5** |
| **12. I felt tense during the game. (R)** | **1 2 3 4 5** |
| **13. I felt unfriendly during the game (R)** | **1 2 3 4 5** |
| **14. I felt sad during the game. (R)** | **1 2 3 4 5** |
| **15. I felt relaxed during the game.** | **1 2 3 4 5** |
| **16. I felt friendly during the game.** | **1 2 3 4 5** |

**Figure S2. Linear regressions**

1. Linear regression between childhood adversity and psychosocial functioning
2. Linear regression between friendship quality and psychosocial functioning


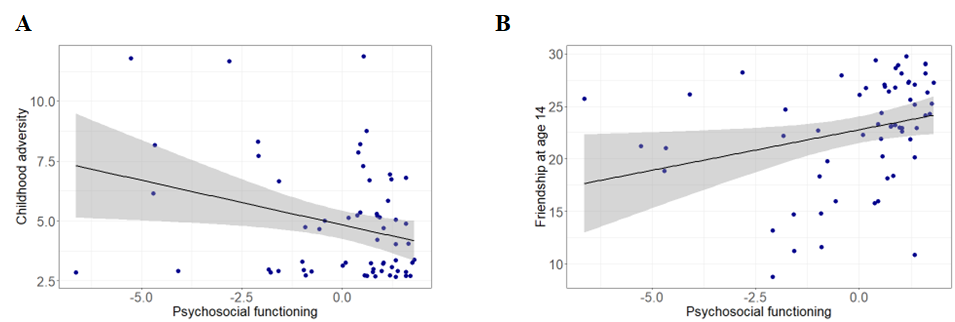


**Figure S3.** Association between friendships at age 14 and mood scores.

**A.** Association between friendships at age 14 and change in mood across both the inclusion and exclusion conditions of the Cyberball task.

**B.** Association between friendships at age 14 and mood after the inclusion condition of the Cyberball task.

**C.** Association between friendships at age 14 and mood after the exclusion condition of the Cyberball task.

**
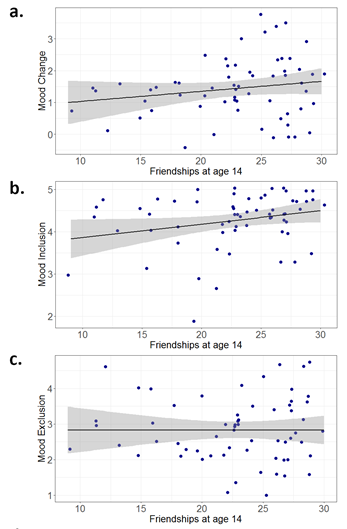
**

**Figure S4.** Brain activations found during ‘*Not receiving the ball during the exclusion condition vs. Receiving the ball during the inclusion condition’* contrast. Participants activated a cluster encompassing the right insula and rolandic operculum (peak at x, y, z = 40, -14, 24, t = 6.29, pFWE < 0.05). No significant activations were found in the contrast *‘Receiving the ball during the inclusion condition vs. Not receiving the ball during the exclusion condition’* at pFWE < 0.05.


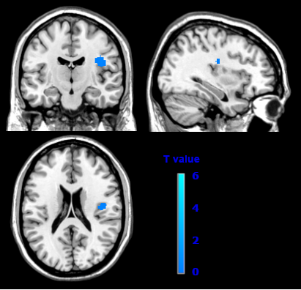


**References**

Iacobucci, D., Saldanha, N., Deng, X., 2007. A Meditation on Mediation: Evidence That Structural Equations Models Perform Better Than Regressions. J. Consum. Psychol. 17, 139–153. https://doi.org/https://doi.org/10.1016/S1057-7408(07)70020-7

Rosseel, Y., 2012. lavaan: An R Package for Structural Equation Modeling. J. Stat. Softw. 48, 1–36.

Sobel, M., 1982. Asymptotic confidence intervals for indirect effects in structural equation models. Sociological methodology. Wiley.
